# A novel prognostic signature for clear cell renal cell carcinoma constructed using necroptosis-related miRNAs

**DOI:** 10.1186/s12864-023-09258-9

**Published:** 2023-03-29

**Authors:** Zuhu Yu, Chong Lu, Bin Lu, Hong Gao, Rongfang Liang, Wuxing Xiang

**Affiliations:** 1 Department of Urology, University of Chinese Academy of Sciences-Shenzhen Hospital, No.4253 SongBai Road, Guangming District, Shenzhen, 518107 China; 2 Anhui Medical University, Hefei, 230032 China; 3 Guangdong and Shenzhen Key Laboratory of Male Reproductive Medicine and Genetics, Peking University Shenzhen Hospital, Shenzhen, 518036 China

**Keywords:** ccRCC, Necroptosis, miRNA, Prognosis

## Abstract

**Background:**

This work aims to analyze the relationship between necroptosis-related microRNAs (miRNAs) and the prognosis of clear cell renal cell carcinoma (ccRCC).

**Methods:**

The miRNAs expression profiles of ccRCC and normal renal tissues from The Cancer Genome Atlas (TCGA) database were used to construct a matrix of the 13 necroptosis-related miRNAs. Cox regression analysis was used to construct a signature to predict the overall survival of ccRCC patients. The genes targeted by the necroptosis-related miRNAs in the prognostic signature were predicted using miRNA databases. Gene Ontology (Go) and Kyoto Encyclopedia of Genes and Genomes (KEGG) analyses were used to investigate the genes targeted by the necroptosis-related miRNAs. The expression levels of selected miRNAs in 15 paired samples (of ccRCC tissues and adjacent normal renal tissues) were investigated using reverse transcriptase quantitative polymerase chain reaction (RT-qPCR).

**Results:**

Six necroptosis-related miRNAs were found to differentially expressed between ccRCC and normal renal tissues. A prognostic signature consisting of miR-223-3p, miR-200a-5p, and miR-500a-3p was constructed using Cox regression analysis and risk scores were calculated. Multivariate Cox regression analysis showed that the hazard ratio was 2.0315 (1.2627–3.2685, P = 0.0035), indicating that the risk score of the signature was an independent risk factor. The receiver operating characteristic (ROC) curve showed that the signature has a favorable predictive capacity and the Kaplan-Meier survival analysis indicated that ccRCC patients with higher risk scores had worse prognoses (P < 0.001). The results of the RT-qPCR verified that all three miRNAs used in the signature were differentially expressed between ccRCC and normal tissues (P < 0.05).

**Conclusion:**

The three necroptosis-related-miRNAs used in this study could be a valuable signature for the prognosis of ccRCC patients. Necroptosis-related miRNAs should be further explored as prognostic indicators for ccRCC.

## Introduction

Clear cell renal cell carcinoma (ccRCC) is the most common histological subtype of renal cell carcinoma that metastasizes easily and patients with ccRCC have poor prognoses [1]. As in the case of most solid malignancies, the mechanism of occurrence, progression, and metastasis of ccRCC remain poorly understood. Patients with ccRCC having localized tumors have relatively good prognoses after surgical resections; however, patients with advanced ccRCC usually do not have very good prognoses even with systemic therapy [2]. The complex etiology and pathophysiology of ccRCC make it difficult to predict therapy outcomes. Since there are limited therapeutic strategies available to treat ccRCC, especially in advanced cases, new prognostic models are needed to help in deciding appropriate treatment strategies.

Necroptosis is a form of programmed cell death, which is mechanistically similar to apoptosis but also presents the morphological characteristics of necrosis. Necroptosis is not initiated via the caspase pathway; instead, it is initiated by receptor-interacting protein kinase 1 (RIPK1), receptor-interacting protein kinase 3 (RIPK3), and mixed lineage kinase domain-like pseudokinase (MLKL). The process is characterized by the early loss of plasma membrane integrity, release of inflammatory factors and organelle swelling [3, 4]. Evidence suggests that necroptosis plays a critical role in inflammatory and non-inflammatory processes associated tumorigenesis, progression, and immune evasion ability of cancers [5]. These discoveries have provided researchers with a new strategy for treating cancers by using drugs that can induce necroptosis.

MicroRNAs (miRNAs) are short (19–25 nucleotides long) endogenous non-coding RNAs found in advanced eukaryotes. Unlike small interfering RNAs (siRNAs), mature miRNAs usually guide RNA-induced silencing complex (RISC) to bind to specific mRNA targets, which leads to the degradation of these mRNAs or repression of translation, ultimately lowering the expression levels of the target genes [6]. The crucial role played by miRNAs in cell growth, metabolism, differentiation, and death has been amply demonstrated [7]. The influence of the miRNAs on oncogenic characteristics such as excessive proliferation, immune evasion, resistance to apoptosis, and metastasis are also been explored [8, 9]. Numerous studies have demonstrated the relationships between miRNAs and prognosis in cancer patients [10, 11]. In ccRCC, several aberrantly expressed miRNAs have been found to participate in tumor proliferation, cell migration, and anti-apoptosis signaling; all of these may affect the prognosis of patients [12–14]. However, few investigations have attempted to use necroptosis-related miRNAs as prognostic markers for patients with ccRCC.

In this work, we used data from public databases to explore the use of miRNA expression profiles in predicting the prognosis of patients with ccRCC. We used a matrix of miRNAs to extract information on those associated with necroptosis. We then constructed a prognostic signature with necroptosis-related miRNAs using variation analysis and univariate and multivariate Cox regression analysis. We also identified the target genes of the prognostic miRNAs using multiple databases and performed a functional enrichment analysis of these target genes to explore how these miRNAs affect tumor growth in ccRCC. We further verified the aberrant expression patterns of three necroptosis-related miRNAs in the signature of ccRCC and normal tissues using reverse transcriptase quantitative polymerase chain reaction (RT-qPCR).

## Materials and methods

### Data acquisition and processing

We downloaded the expression profiles of miRNAs and the relevant clinical information on ccRCC patients from The Cancer Genome Atlas (TCGA) database (https://portal.gdc.cancer.gov/), including the raw counts of the miRNAs from 72 normal renal tissues and 539 tumor samples. We have previously identified 13 necroptosis-related miRNAs which were associated with cancer metastasis from Pubmed [15]. Subsequently, the expression matrix of the 13 necroptosis-related miRNAs and related clinical data was extracted, matched, filtered, and revised for further analysis. The patients’ clinical data included their gender, age, survival time (> 30 days), survival state, and the grade, stage, and TNM classification of their ccRCC tumors.

### Construction of a prognostic signature with necroptosis-related miRNAs

The ‘limma’ package in R (version 4.1.1) was used to identify the miRNAs that are differentially expressed between the normal and ccRCC tissues with a filtering condition of false discovery rate (FDR) < 0.05. The results of this analysis are represented as a heatmap. Following this, we used univariate and multivariate Cox regression to determine if one or more of the necroptosis-related miRNAs were associated with prognoses in ccRCC. The results are represented in a forest plot. The miRNAs that were significantly (P < 0.05) associated with prognosis were identified and used to construct a prognostic signature and risk score for patients. In addition, univariate and multivariate Cox regression analyses were used to determine if the risk score was an independent risk factor when integrating clinical characteristics. The predictive ability of the signature was assessed using the area under curve (AUC) statistic from the receiver operating characteristic (ROC) curve. Following this analysis, all patients were divided into low-risk or high-risk groups as per the median value of the risk score. The Kaplan-Meier curves of joint and individual miRNA signatures were plotted to further determine the relationship between the necroptosis-related miRNAs and the prognosis of patients with ccRCC. The packages ‘survival’, ‘ROCR’, and ‘timeROC’ in R were used in these analyses.

### Gene ontology (GO) and kyoto encyclopedia of genes and genomes (KEGG) analysis of target genes of necroptosis-miRNAs

Three miRNA databases—miRDB, TargetScan, and miTarBase—were used to identify the genes targeted by the necroptosis-related miRNAs that were significantly correlated to the prognosis of patients with ccRCC [16–18]. The intersecting genes obtained from these databases were identified as targets for the necroptosis-related miRNAs. Cytoscape (version3.9.0) was used to demonstrate the miRNA and target gene interaction network. The package ‘clusterProfiler’ in R was used to carry out the GO and KEGG analyses on the target genes [19–23].

### RNA extraction and RT-qPCR

The ccRCC and paired normal renal tissues were collected from 15 patients who were diagnosed with ccRCC at the Peking University Shenzhen Hospital. Informed consent was obtained from all patients. This study was approved by the Ethics Committee of the Peking University Shenzhen Hospital. Total RNA from each sample was extracted with a TRIzol LS isolation kit (Thermo Fisher Scientific, USA) and stored at − 80 °C according to the manufacturer’s instructions. The RT-qPCR was performed using a SYBR Green qPCR Kit (SYBR Pre-mix Ex Taq II, TaKaRa, Japan) and the LightCycler480 Real-Time PCR system (Roche Diagnostics, Germany). The parameters of the reaction were set to: denaturation at 95 °C for 20 s, followed by 40 cycles of denaturation at 95 °C for 15 s, annealing at 60 °C for 45 s, and then extension at 60 °C for one minute. The U6 RNA was used as an internal control.

### Statistical analysis

The expression levels of the necroptosis-related miRNAs were calculated using the formula 2^−ΔΔCt^ method after normalization with the internal control RNA. The Student’s t-test in GraphPad Prism (Version 9.4.0) was used to compare the expression levels of the miRNAs in the ccRCC and adjacent normal renal tissues. Results with P-value < 0.05 were considered statistically significant.

## Results

### Necroptosis-related miRNAs that are differentially expressed between ccRCC and normal tissues

**Fig. 1:**
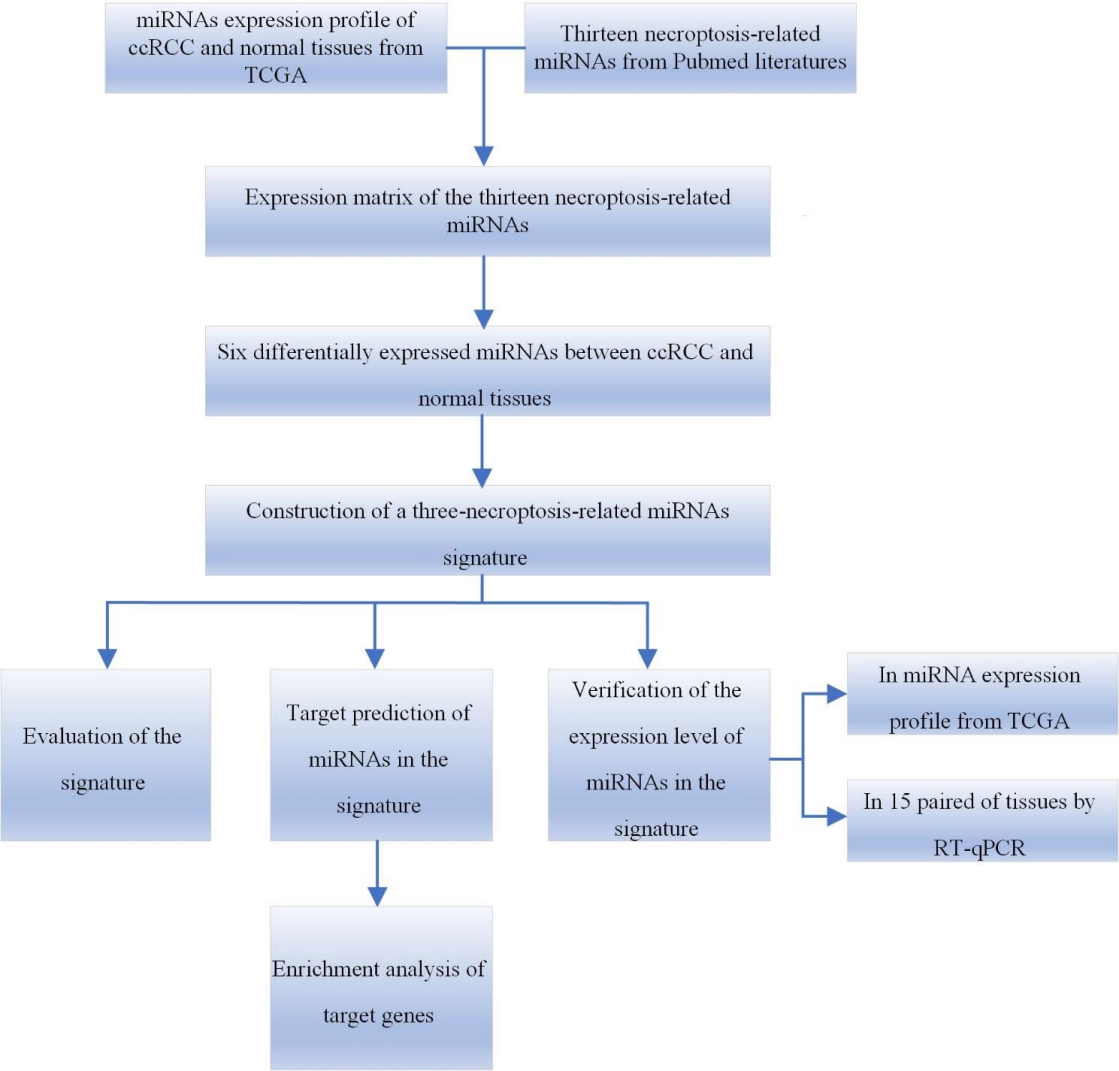
The flowchart of the study

**Fig. 2:**
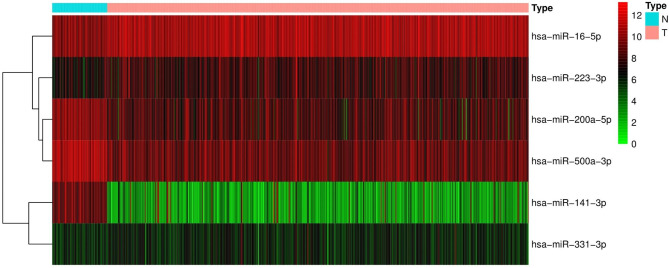
The heatmap of the six miRNAs that are differentially expressed between ccRCC and normal renal tissues

Figure 1 shows a flowchart indicating the various steps of this study. Data on the expression levels of 13 miRNAs from ccRCC and normal tissues were extracted and analyzed. We identified six miRNAs (Fig. 2) which were differentially expressed between ccRCC and normal renal tissues, namely, miR-16-5p (logFC = 0.1258, FDR < 0.001), miR-223-3p (logFC = 0.1738, FDR < 0.001), miR-200a-5p (logFC=-0.2835, FDR < 0.001), miR-500a-3p (logFC=-0.2622, FDR < 0.001), miR-141-3p (logFC=-1.4583, FDR < 0.001) and miR-331-3p (logFC = 0.0605, FDR = 0.015).

### Prognostic signature of necroptosis-related miRNA

**Fig. 3:**
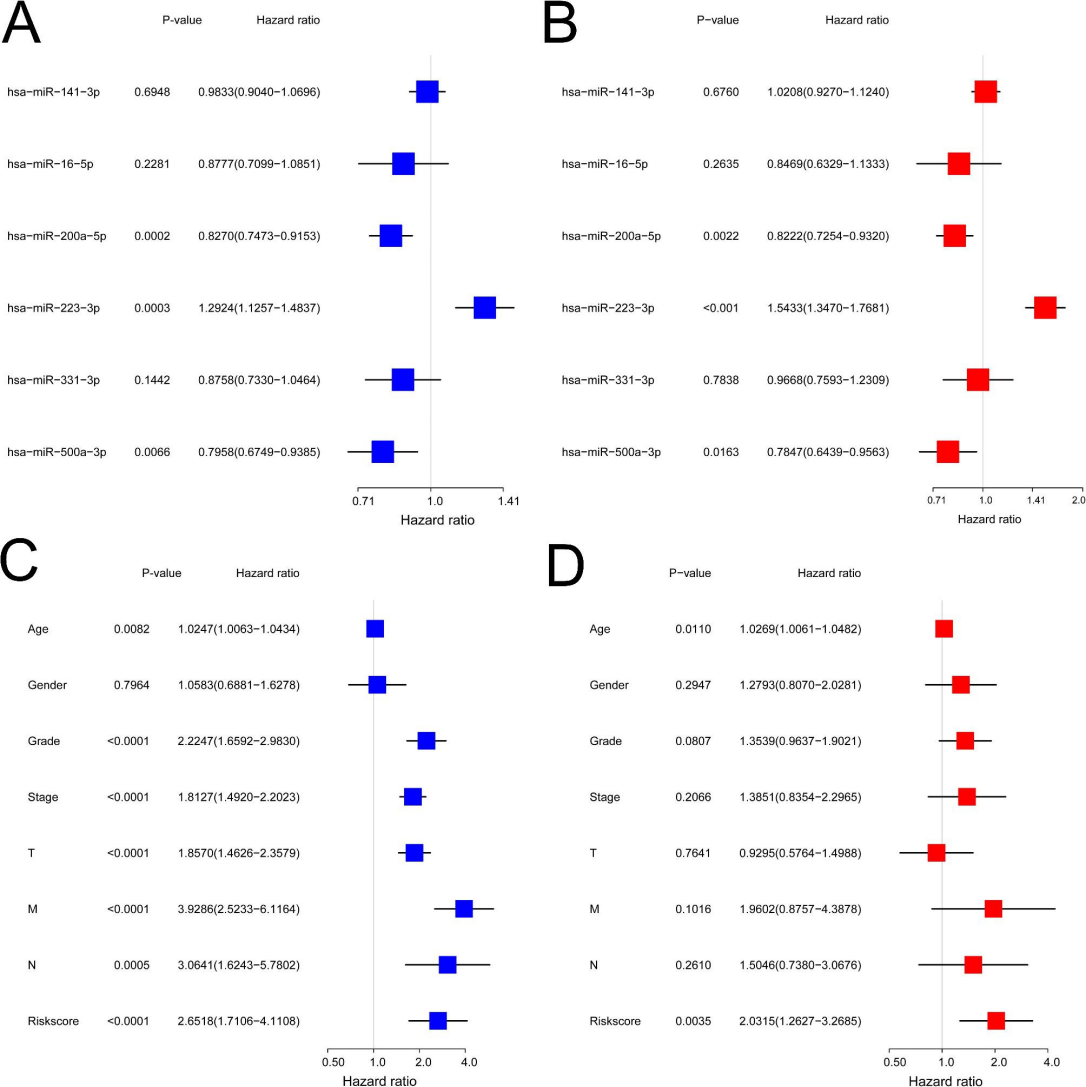
Construction of the prognostic signature. **(A)** The univariate Cox regression analysis of the six differentially expressed miRNAs indicates that has-miR-200a-5p, has-miR-223-3p, and has-miR-500a-3p were correlated with the survival times of patients with ccRCC (P < 0.05). **(B)** The results of multivariate Cox regression analysis of the six differentially expressed miRNAs are similar to those of the univariate analysis (P < 0.05). **(C)** The univariate Cox regression analysis of the risk score and clinical characteristics reveals that the tumor grade, tumor stage, and TNM classification were correlated with overall survival (P < 0.001). **(D)** The multivariate Cox regression analysis of the risk score and clinical characteristics indicates that the risk score is an independent prognostic factor (P = 0.0035)

Outcomes of univariate and multivariate Cox regression analyses of the six miRNAs are shown in Fig. 3A and B. Based on these results, we chose miR-200a-5p, miR-223-3p, and miR-500a-3p to construct the prognostic signature and risk score as per the formula given below:

risk score = (0.4039 × miR-223-3p_exp_) − (0.2094 × miR-200a-5p_exp_) − (0.2734 × miR-500a-3p_exp_)

Following this, we used a Cox regression analysis to assess the value of the risk score as an independent prognostic factor when combined with other clinical characteristics, namely, age, gender, tumor grade, tumor stage, and TNM stage. The results showed that the risk score is an independent risk factor and that higher risk score indicated a worse prognosis for patients with ccRCC (P < 0.05). As shown in Fig. 3 C and 3D, the hazard ratios of the univariate and multivariate Cox regression analyses were 2.6518 (1.7106–4.1108, P < 0.0001) and 2.0315 (1.2627–3.2685, P = 0.0035), respectively.

**Fig. 4:**
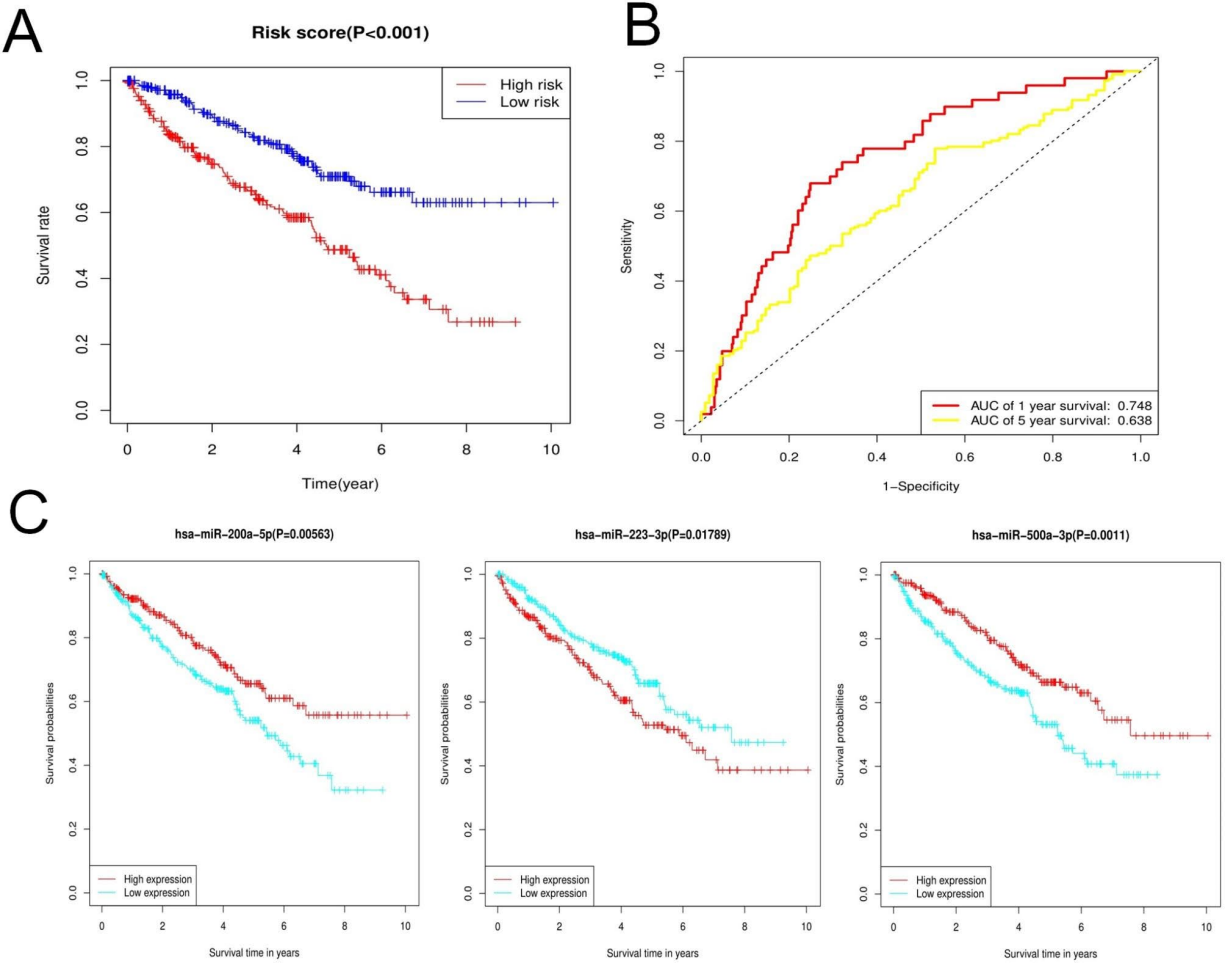
Evaluation of the constructed signature. **(A)** The Kaplan-Meier survival analysis demonstrats that patients with higher risk scores had worse prognoses (P < 0.001). **(B)** The AUC of the 1-year survival and 5-year survival curves indicated that the signature had a good predictive capacity. **(C)** The survival analysis of miRNAs in the signature shows that high expression levels of miR-223-3p and low expression levels of miR-200a-5p and miR-500a-3p were associated with poor prognoses (P < 0.05)

The Kaplan-Meier survival analysis of the risk score also indicated that higher risk scores indicated worse prognosis for patients with ccRCC (P < 0.001, Fig. 4A). The AUC for the 1-year survival curve was 0.748 and that for the 5-year survival curve was 0.638, demonstrating that this signature had good predictive value (Fig. 4B). To further illustrate the correlation between the necroptosis-related miRNAs and prognosis, we carried out survival analysis for each miRNA in the signature. The results revealed that all the three miRNAs used in the signature were significantly correlated with the prognosis; the patients with low expression levels of miR − 200a − 5p (P = 0.0056) and miR − 500a − 3p (P = 0.0011) in the tumor tissues had unfavorable prognoses whereas low expression levels of miR-223-3p (P = 0.0178) in the tumor tissues was associated with favorable prognoses (Fig. 4C).

### Results of the enrichment analysis of the target genes

**Fig. 5:**
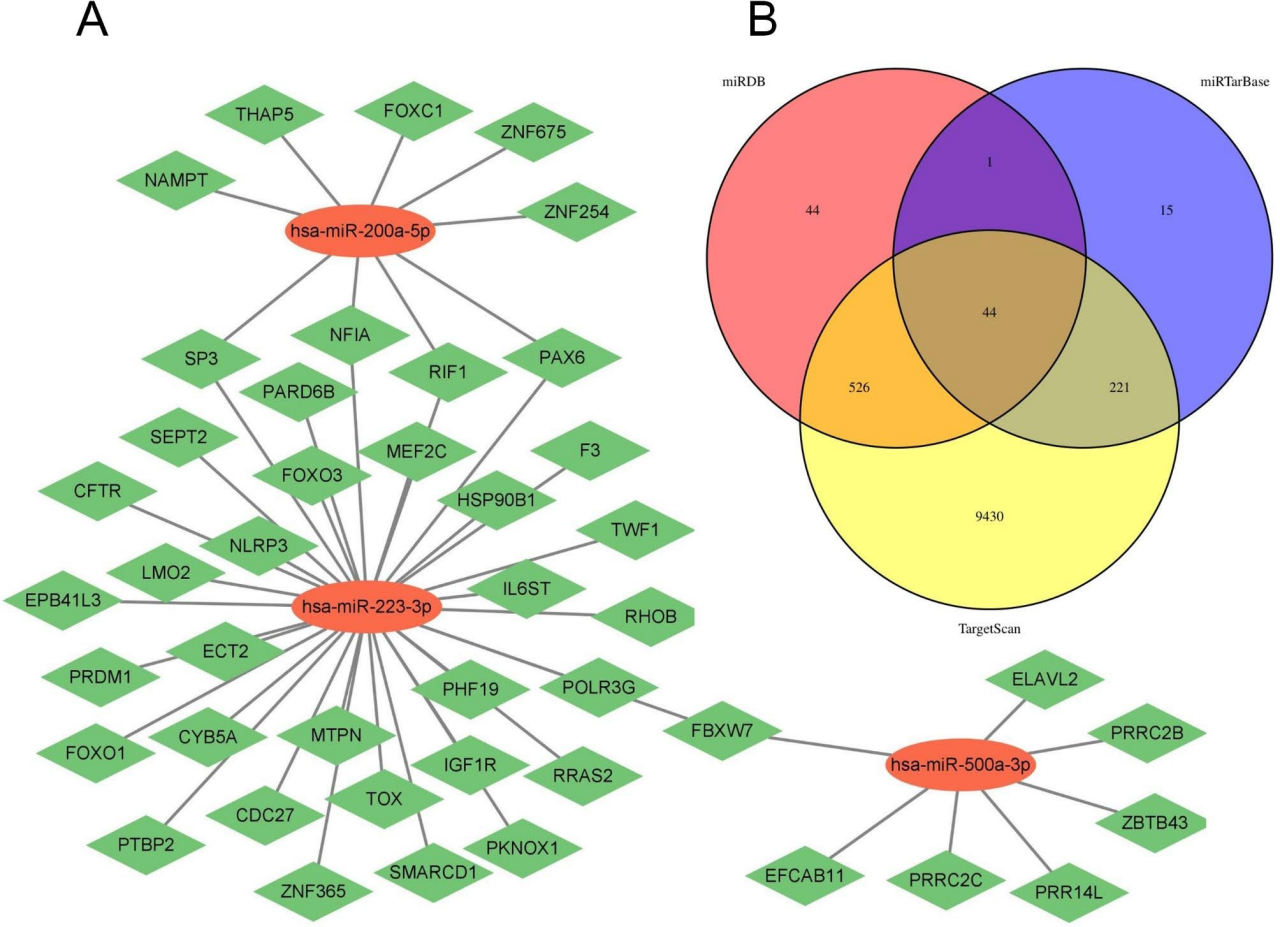
Screening for genes targeted by the necroptosis-related miRNAs in the signature. **(A)** Interaction network of the miRNAs and their target genes. **(B)** Venn diagram of the target genes

**Fig. 6:**
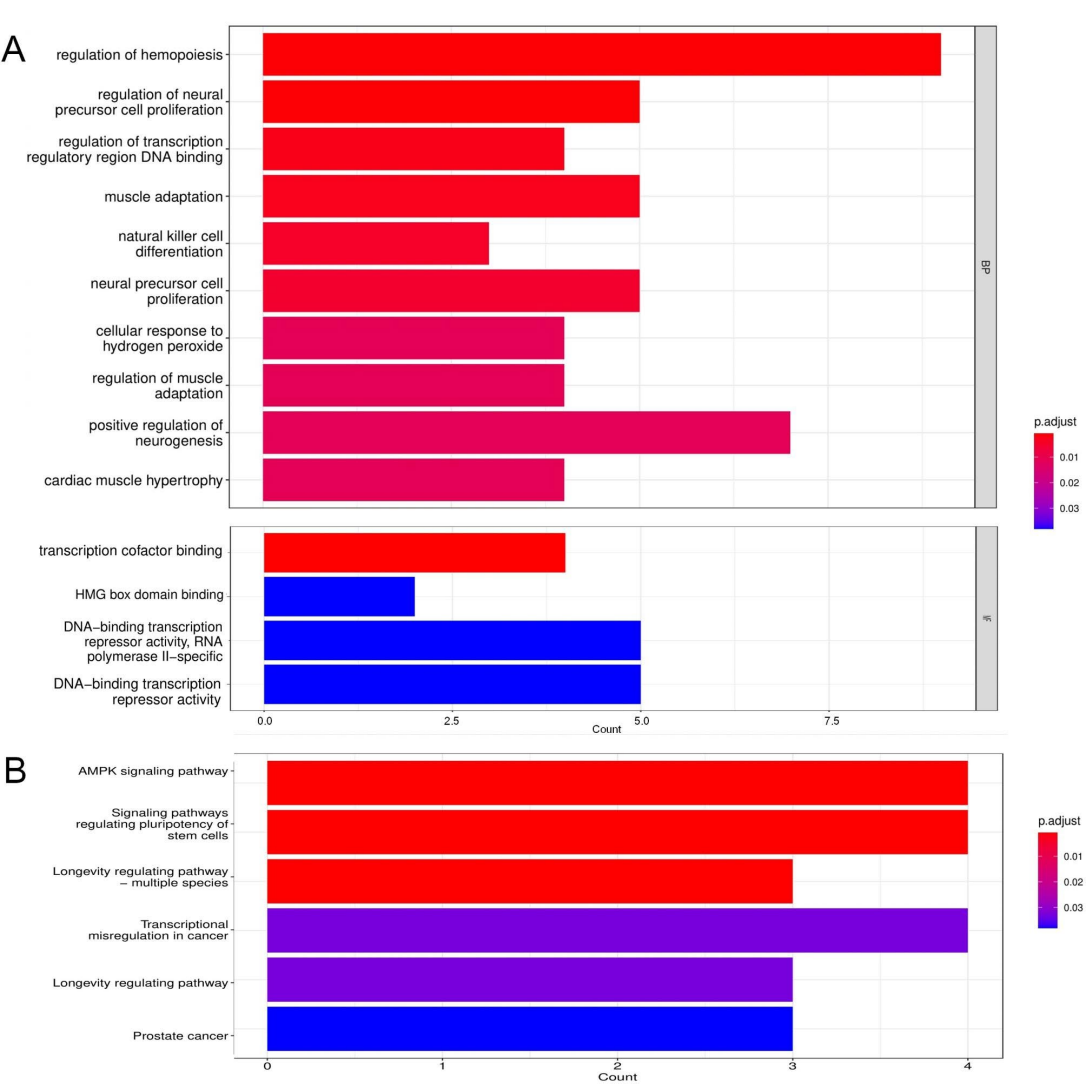
Enrichment analysis of the target genes. **(A)** Results of the GO analysis of the target genes. **(B)** Results of the KEGG analysis of the target genes

We used three miRNA databases to predict the genes targeted by the three miRNAs used in this study and identified a total of 44 target genes (Fig. 5B). The miRNA and target gene interaction network was constructed with Cytoscape, as shown in Fig. 5A, followed by GO and KEGG analyses of the 44 target genes (Fig. 6). The GO analysis indicated that the target genes were enriched in genes associated with the regulation of hemopoiesis, neural precursor cell proliferation, transcription regulatory region DNA binding, muscle adaptation, natural killer cell differentiation, neural precursor cell proliferation, cellular response to hydrogen peroxide, transcription cofactor binding, high mobility group (HMG) box domain binding, and DNA-binding transcription repressor activity. KEGG analysis revealed that the target genes were enriched in pathways such as the adenosine monophosphate-activated protein kinase (AMPK) signaling pathway, signaling pathways regulating the pluripotency of stem cells, longevity regulating pathway-multiple species, and transcriptional misregulation in cancer.

### Expression levels of the three selected miRNAs in ccRCC and normal renal tissues

**Fig. 7:**
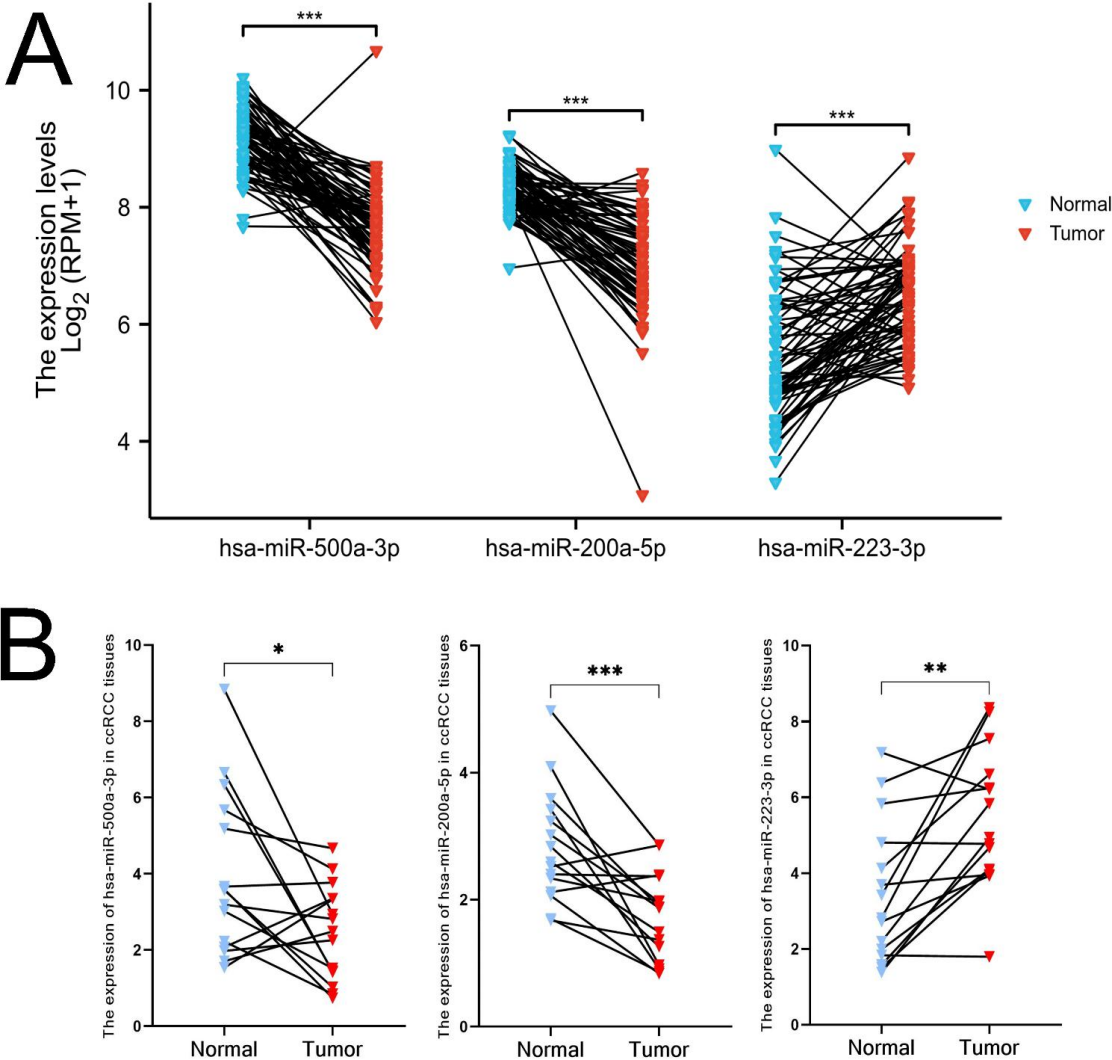
Verification of the expression levels of three necroptosis-related miRNA. (A) The expression levels of the three necroptosis-related miRNAs in ccRCC and adjacent normal renal tissues were analyzed using the TCGA database. (B) The expression levels of the three necroptosis-related miRNAs in the 15 paired of samples (ccRCC and adjacent normal tissues) as verified by RT-qPCR. (*P < 0.05, **P < 0.01, ***P < 0.001)

We analyzed the expressions levels of the three miRNAs used in the prognostic signature that we have developed from data obtained in the TCGA database (Fig. 7A). We further verified the expressions of these three miRNAs in paired samples (ccRCC and adjacent normal renal tissues) using RT-qPCR (Fig. 7B). The results indicated that the expression levels of miR-500a-3p and miR-200a-5p are significantly lower, and that of miR-223-3p is significantly higher in ccRCC tissues as compared to those in normal tissues (P < 0.05). These results also support the results of the survival analysis of each miRNA in the signature.

## Discussion

As the most common subtype of renal cancer, ccRCC is responsible for the majority of deaths caused by kidney cancer. Approximately 30% of all patients with localized ccRCC suffered recurrence and metastasis despite treatment [24]. Although it is well known that mutations in genes like Von Hippel-Lindau (VHL) associated with the development of ccRCC, and there are many RNA and protein markers associated with prognosis in patients with ccRCC, currently, there is no reliable prognostic biomarker which can be used in clinical practice [25, 26].

Although there has been much research on the use of miRNAs as a prognostic marker for ccRCC, few considered the use of necroptosis-related miRNA [14]. In this work, we have analyzed the expression of necroptosis-related miRNAs using data from the TCGA database, and have identified six miRNAs that are differentially expressed between ccRCC and normal renal tissues. We selected three of these miRNAs (miR-223-3p, miR-200a-5p, miR-500a-3p) and constructed a prognostic signature for ccRCC. Further analysis proved that the signature is an independent biomarker for the prognosis of patients with ccRCC. Survival analysis and validation of the expression levels of these miRNAs in paired samples (of ccRCC and normal renal tissues) indicate that the signature and all the three miRNAs used in the signature were significantly associated with the prognosis of patients with ccRCC.

Bao et al. (2022) have constructed a signature with six necroptosis-related miRNAs as a prognostic indicator for patients with ccRCC [27]; the miRNAs used in their signature included miR-193a-3p, miR-101-3p, miR-200a-5p, miR-214-3p, miR-221-3p and miR-223-3p, which partially overlapped with the signature that we have developed and described in this work. However, after verification of the miRNA expression levels using 12 paired samples (of ccRCC and adjacent normal tissues), only two of the miRNAs (miR-193a-3p and miR-214-3p) were found to be significantly differentially expressed. In our study, we have demonstrated that the miR-223-3p, miR-200a-5p, and miR-500a-3p are differentially expressed between ccRCC and normal renal tissues. The AUCs of the 1- and 5-year survival curves of the signature constructed by Bao et al. (2022) were 0.721 and 0.703, and the hazard ratios of the risk score and stage in univariate Cox regression analysis were 2.9710 (1.8281–4.8286, P < 0.0001) and 3.6102 (2.2779–5.7216, P < 0.0001), respectively. In our signature, the AUCs of the 1- and 5-year survival curves were 0.748 and 0.638, and univariate Cox regression analysis showed that the hazard ratios of risk score and stage were 2.6518 (1.7106–4.1108, P < 0.0001) and 1.8127 (1.4920–2.2023, P < 0.0001), respectively. A comparison between the two signatures shows that the prognostic performance of our signature is equivalent to the signature developed by Bao et al. (2022). However, our signature is more concise and economical as fewer miRNAs are used; this is especially important for clinical practice as both, the time taken and the cost of obtaining the information for prognostic signatures are important factors.

Prior work has confirmed that necroptosis is involved in inflammatory, neurodegenerative, ischemic cardiovascular, and cerebrovascular diseases, as well as in cancers [5]. However, necroptosis can play a dual role in cancers [28, 29]. On one hand, necroptosis can inhibit the progression of some cancers by functioning as an alternative pathway for cell death when apoptosis fails, however, on the other hand, necroptosis can also contribute to inflammatory reactions and immunosuppression in some cancers. Recently, many necroptosis-related miRNA signatures have been constructed using computational methods to predict the prognosis of several cancers such as intestinal cancer, lung adenocarcinoma, breast cancer, and gliomas [30–33]. Compared to traditional experiments, computational methods are significantly quicker and cheaper to perform and demonstrate excellent efficiency in the establishment of prognostic models for complex human diseases [34, 35]. Although it has become a trend to develop prognostic markers for cancers by using databases and computational methods, experimental verifications are still required to gauge the effectiveness of these prognostic signatures [36].

Previous studies have confirmed that miR-223-3p suppresses necroptosis in myocardial ischemia/reperfusion injury, acute kidney injury induced by 3-chloro-1,2-propanediol (3-MCPD)-dipalmitate, and spinal cord injury, by targeting the cell death receptors in necroptotic pathway and the inflammatory response [37, 38]. In kidney injuries, miR-500a-3p is thought to mitigate the toxicity and ischemic damage caused by necroptosis and inflammatory responses by targeting the MLKL and RIPK3 proteins according to experiments in HK2 cells [39]. Another miRNA used in this study, miR-200a-5p, is known to prevent myocardial necroptosis induced by selenium deficiency [40]. In melanomas, papillary thyroid carcinomas, ovarian carcinomas, and lung cancers, miR-200a-5p promotes cancer by inhibiting necroptosis [41–44]. However, miR-200a-5p has the opposite effect in breast cancers as suppresses cell proliferation by regulating the expression of hepatocyte growth factor (MET) and epidermal growth factor receptor (EGFR) [45].

In this work, we have constructed a novel prognostic signature with necroptosis-related miRNAs to predict the prognosis of patients with ccRCC. This signature used only three miRNAs but showed excellent prognostic capacity which makes it easier to use and more economical than the previously developed signature that used six miRNAs. However, this study has several limitations. One limitation is that only TCGA datasets were; used in this study more datasets from other databases are needed for the external validation of the signature. Two, the applicability of the signature needs to be validated with robust clinical studies from multiple medical centers before it can be used in regularly in clinical practice. Third, the specific functions and mechanisms of how these necroptosis-related miRNAs affect the occurrence and progression of ccRCC need to be carefully explored.

## Conclusions

We have constructed a novel prognostic signature using necroptosis-related miRNAs which can be used as an independent marker for predicting the prognosis of patients with ccRCC. The three necroptosis-related miRNAs used to construct the signature showed excellent prognostic capacity and provided a foundation for using necroptosis-related miRNAs as prognostic marker in renal cancer.

## Data Availability

The RNA-seq data and clinical information in this study are available in the TCGA database (https://portal.gdc.cancer.gov/). The details of the data used in this manuscript are available from the corresponding author.
